# Efficient Tuning of the Third-Order Nonlinear Optical Properties of Some Functionalized Boron-Dipyrromethene Dyes

**DOI:** 10.3390/nano15201549

**Published:** 2025-10-11

**Authors:** Ioannis Orfanos, Panagiotis Aloukos, Antonia Kaloudi-Chantzea, George Pistolis, Stelios Couris

**Affiliations:** 1 Department of Physics, University of Patras, 26504 Patras, Greece; 2 Institute of Nanoscience and Nanotechnology, National Centre for Scientific Research Demokritos, 15310 Athens, Greece; tonia.kaloudi@gmail.com (A.K.-C.);

**Keywords:** Boron–Dipyrromethene derivatives, BODIPYs, nonlinear optical properties, Z-scan, hyperpolarizability, saturable absorption

## Abstract

In the present work, the third-order nonlinear optical (NLO) response of some recently synthesized, functionally substituted Boron–Dipyrromethene (BODIPY) derivatives is studied, and their nonlinear optical properties are investigated using the Z-scan technique, employing 4 ns, visible (532 nm) and near-infrared (1064 nm) laser excitation. The present findings demonstrate the importance of functionalization of the BODIPY core and the versatility it offers for the modification of the photophysical properties of these chromophores, allowing for the very efficient tuning of both the magnitude and the sign of the nonlinear absorption and refraction of the BODIPYs, making them very promising materials for several optoelectronic applications. The obtained results are discussed and compared with the results of other similar BODIPYs reported in the literature.

## 1. Introduction

Boron–Dipyrromethene dyes (BODIPYs) have emerged as a versatile and highly promising class of organic compounds due to their exceptional photophysical and spectroscopic properties. Over the recent years, extensive research has highlighted their potential in numerous scientific and technological applications [1,2,3,4]. These small-molecule fluorophores exhibit strong fluorescence with remarkably high quantum yields (Φ), properties which can be systematically tuned across the visible to near-infrared spectrum through structural modifications of either their indacene core or the BODIPY’s framework [1,2,3,4]. This tunability, extending beyond the emission and absorption characteristics, provides an adaptable platform for a wide range of optical functionalities. An important advantage of BODIPYs lies in their unique photostability and chemical robustness under varying environmental conditions, such as changes in polarity or pH, qualities that make them particularly attractive for sensor applications [1,2,3]. The chemical versatility of BODIPYs is further enhanced by their amenability to multiple, site-specific substitutions, which allows fine-tuning of their electronic and optical properties through tailored functional groups [1,5,6]. This enables the extension of the π-conjugation system or exocyclic modification, leading to substantial shifts in absorption and emission wavelengths and facilitating color tunability over a broad range [2].

Another important feature of the BODIPY family is their capability to act as electron donors when paired with suitable electron acceptors, engaging in light-induced electron transfer processes either via through-bond or through-space mechanisms [7]. This property underpins their utility in the construction of complex conjugated systems, including covalently linked porphyrin and fullerene conjugates [8,9], as well as self-assembled multichromophoric arrays based on BODIPY subunits [10]. These supramolecular architectures have attracted considerable interest for their light-harvesting capabilities, making them very promising candidates for advanced photonic and photovoltaic materials.

Consequently, the broad range of photophysical and chemical features of BODIPYs has rendered them valuable in diverse applications, including chemical sensing [11], photodynamic therapy [12], bioimaging [13], organic photovoltaics [14], organic electronics [15], and laser technologies [16]. The pursuit of novel broadband laser sources with improved operational stability, extended lifetimes, and enhanced performance characteristics relative to conventional organic dyes such as cyanines [1], squaraines [2], porphyrins [3], phthalocyanines [17], azobenzenes [18], and metal dithiolenes [19] has driven particular interest towards BODIPY derivatives. Their large emission and absorption tunability, coupled with superior photo- and thermal stability, position BODIPYs as promising candidates for both laser dyes and organic solid-state laser materials [16]. Within this context, numerous studies have focused on the nonlinear optical (NLO) properties of BODIPYs, emphasizing the role of intramolecular charge transfer (CT) in modulating their NLO response [20,21,22]. In general, increasing the conjugation length or enhancing the electron-donating strength of substituents leads to significant amplification of second- and third-order NLO responses. For instance, a variety of BODIPY derivatives have been reported to exhibit large two-photon absorption (TPA) cross-sections and effective optical limiting action at telecommunications-relevant wavelengths [23]. Furthermore, investigations into singlet–triplet intersystem crossing have revealed substituent-dependent quantum yields and triplet-state lifetimes typically on the microsecond scale, which are relevant for photodynamic and various optoelectronic applications [24]. The formation of intramolecular CT states has also been established in aza-BODIPYs, with their formation strongly influenced by solution basicity [25]. Such CT states are critical contributors to the NLO response of these molecules, in line with observations in other molecular and supramolecular systems. Examples include ferrocene– and [60]fullerene–porphyrin dyads, where charge-separated states notably enhance the NLO response [26,27,28]. This underlines the importance of charge separation dynamics in optimizing the optical performance of BODIPY-based materials.

The present study investigates the NLO response of four newly synthesized functionally substituted BODIPY derivatives using the Z-scan technique, and employing 4 ns, visible (532 nm) and infrared (1064 nm) laser excitation. The BODIPY core unit has also been studied under the same experimental conditions for comparison purposes. The obtained results are analyzed, discussed, and compared with the other literature data of similar structured BODIPY compounds.

## 2. Experimental Section

### 2.1. Materials

**Scheme 1:**
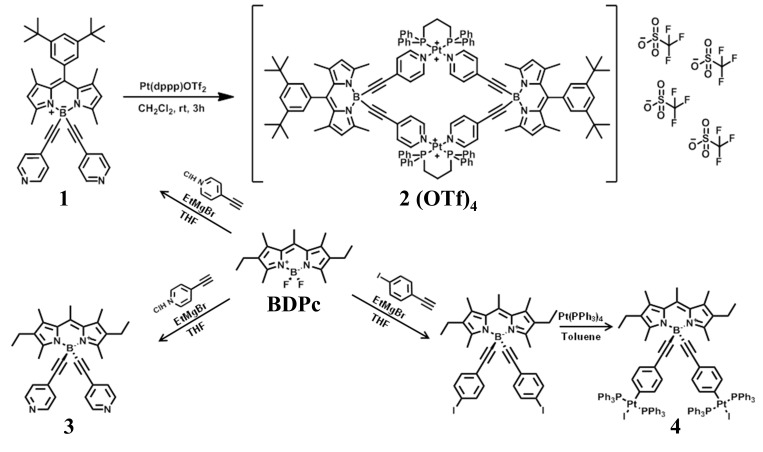
Schematic illustration of the synthesis of the Boron–Dipyrromethene (BODIPY) dyes studied. Details on the synthetic procedure are given elsewhere: compound **1** and its supramolecular assembly **2(OTf)_4_** in ref. [31], **BDPc** and **3** in ref. [29], and **4** in ref. [30].

The details of the synthesis can be found elsewhere [29,30,31]. The synthesis of compound **1** and its coordination-driven self-assembly, with a 90° organoplatinum acceptor to form the intensely fluorescent rhomboid cavitand **2(OTf)_4_**, has been reported by some of us elsewhere [31]. Similarly, the synthesis of the basic BODIPY unit, **BDPc**, and its functionalized derivative, **3**, is described in reference [29]. The synthetic procedure for compound **4** can be found in the reference [30] (Scheme 1).

### 2.2. UV-Vis-NIR Absorption Spectra

**Figure 1:**
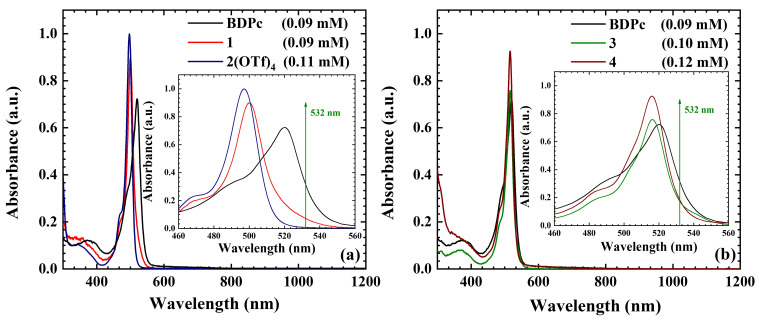
UV-Vis-NIR absorption spectra of the Boron–Dipyrromethene (BODIPY) derivatives (**a**) BODIPYs BDPc, **1** and **2(OTf)_4 _**and (**b**) BODIPYs BDPc, **3** and **4**.

The BODIPYs studied in this work follow, in general, the typical photophysical characteristics of the regular BODIPY unit, such as high extinction coefficients (i.e., ~10^5^ M^−1^cm^−1^), sharp absorption–fluorescence spectra, associated with a mirror-image relationship, and bright photoluminescence emanated from the lowest π-π* singlet excited-state with quantum yields approaching unity. For their study, several different concentration solutions in dichloromethane (DCM) were prepared, and their UV-Vis-NIR absorption spectra were obtained using a dual-beam UV-Vis-NIR spectrophotometer. In Figure 1, some representative UV-Vis-NIR absorption spectra of the BODIPY derivatives are presented. To make comparisons easier, they are presented in two groups, based on the spectral position of their characteristic absorption band, namely BODIPYs **1** and **2(OTf)_4_** in Figure 1a, and BODIPYs **3** and **4** in Figure 1b. For completeness, the absorption spectrum of **BDPc** is also added in these figures, while the insets present the enlarged views of the spectra around the strong absorption bands of the BODIPYs, with the arrows indicating the laser excitation wavelength at 532 nm. As shown, they all exhibit a characteristic strong absorption band, with its maximum absorption lying between 498 and 520 nm, while they are structureless at longer wavelengths. As can be seen, in the case of **3** and **4**, the laser excitation occurs very close to the maximum of their characteristic strong absorption band, implying practically resonant excitation conditions.

In Table 1, the molecular weight MW, the wavelength, λ^abs^_max_, of the maximum of the characteristic absorption band, the corresponding extinction coefficient, ε_max_, the fluorescence quantum yield, Φ_f_, the wavelength, λ^fl^_max_, of the maximum of fluorescence, the fluorescence radiative rate, k_f_, and the fluorescence lifetime, *τ*, of the studied BODIPYs are summarized. As can be seen from this table, all BODIPYs studied in the present work exhibit very similar photophysical properties.

### 2.3. Measurements of the Nonlinear Optical (NLO) Properties

For the investigation of the NLO properties of the BODIPYs, the Z-scan technique was used, employing a 4 ns Q-switched Nd:YAG laser, operating either at its fundamental at 1064 nm or at its second harmonic output at 532 nm, at a repetition rate of 10 Hz. For the accurate determination of the NLO parameters (i.e., nonlinear absorption coefficient β, and nonlinear refractive index parameter γ′), different concentration solutions of each BODIPY placed in 1 mm thick quartz cells were measured, using several different incident laser intensities. In all cases, the laser beam was focused into the sample by means of a 20 cm plano-convex quartz lens. The beam waist (i.e., half width at 1/e^2^ of irradiance maximum) at the focus was determined using a CCD camera, and it was found to be (18 ± 5) and (31 ± 5) μm for the 532 and 1064 nm laser beams, respectively. The Z-scan technique was selected for the investigation of the NLO properties of the BODIPYs due to its experimental simplicity and because it can provide both the magnitude and the sign of the NLO absorption and refraction from a single measurement. Since a detailed description of the details of the Z-scan technique and the data analysis procedures has been described elsewhere [32], only a brief description will be given in the following for completeness. So, using the Z-scan technique, the variation in the transmittance of a sample is measured as it moves along the propagation direction (e.g., the z-axis) of a focused laser beam, thus experiencing variable laser intensity at each z-position. The variation in the sample’s transmittance is measured simultaneously by two different experimental configurations, the so-called “open-aperture” (OA) and “closed-aperture” (CA) Z-scan ones. In the former, the total transmitted through the sample laser beam is collected and measured by a photodetector (e.g., a photomultiplier), while in the latter, a part of the laser beam, after it has passed through a small pinhole placed in the far-field of the focusing lens, is collected and measured by another photodetector electrically matched to the previous one. Under negligible or low nonlinear absorption conditions, the nonlinear absorption coefficient β and the nonlinear refractive index parameter γ′ can be obtained from the so-obtained OA and CA Z-scans, respectively. In the presence of strong nonlinear absorption, its effect on the recorded CA Z-scan can be removed by dividing the CA by the OA Z-scans, resulting in the so-called “divided” Z-scan, the latter allowing for the determination of the nonlinear refractive parameter γ′.

In particular, the nonlinear absorption coefficient *β* is determined by fitting the OA Z-scan with the following equation:

(1) $$T=\frac{1}{\sqrt{\pi }\left[\frac{\beta \;I_{0}\;L_{eff}}{\left(1+z^{2}/z_{0}^{2}\right)}\right]}\int_{-\infty }^{+\infty }\ln\left[1+\frac{\beta \;I_{0}\;L_{eff}}{\left(1+z^{2}/z_{0}^{2}\right)}\exp\left(-t^{2}\right)\right]\;dt$$

where $L_{eff}=\left[1-\exp\left(\alpha_{0}L\right)\right]/\alpha_{0}$ is the effective sample thickness; $\alpha_{0}$ is the absorption coefficient at the laser excitation wavelength; *L* is the sample length; *Ι*_0_ is the laser peak irradiance; *z*_0_ is the Rayleigh length; and *z* is the position of the sample.

Accordingly, the nonlinear refractive index parameter *γ*′ can be determined from the following relation:

(2) $$\gamma^{\prime }=\frac{\lambda \alpha_{0}}{1-e^{-a_{0}L}}\frac{\Delta T_{p-v}}{0.812\pi I_{0}{\left(1-S\right)}^{0.25}}$$

where Δ*Τ_p-v_* is the total variation in the normalized transmittance obtained from the CA or “divided” Z-scans; $S=1-exp\left(-2{r_{a}}^{2}/{w_{a}}^{2}\right)$ is the aperture linear transmittance, with *r_a_* and *w_a_* being the aperture and beam radii, respectively, and *α*_0_, *L*, and *I*_0_ as defined previously.

After having determined the β and γ′, the imaginary (*Imχ*^(3)^) and real (*Reχ*^(3)^) parts of the third-order nonlinear susceptibility *χ*^(3)^, respectively, can be easily deduced using the following relations:

(3) Imχ3esu=10−7c2 n0296π2ωβcm W−1

(4) Reχ3esu=10−6c n02480 π2γ′cm2 W−1

where c is the speed of light in cm/s, ω is the excitation frequency in *s*^−1^, and *n*_0_ is the linear refractive index.

At this point, it is worth noting that since the third-order susceptibility *χ*^(3)^ is a macroscopic quantity, depending on the concentration of the solute, often, the second hyperpolarizability *γ* is preferred, as it is a molecular constant, describing the NLO response per molecule, therefore allowing for more direct comparisons. *γ* can be calculated from *χ*^(3)^ using the following relation:

(5) $$\gamma =\frac{\chi^{\left(3\right)}}{N\;L^{4}}$$

where *N* is the number of molecules/cm^3^ and $L=\left(n_{0}^{2}+2\right)/3$ is the Lorenz–Lorentz local field correction factor.

From the fitting of the obtained OA Z-scans, the NLO absorption coefficient *β* of each solution was determined, while from the slopes of the curves showing the variation in the Δ*Τ_p-v_* parameter with the peak irradiance, the corresponding NLO refractive index parameter *γ*′ was determined. The NLO response of the solvent (i.e., DCM), measured separately, under identical experimental conditions, was found to be negligible under both excitation regimes (i.e., at 532 and 1064 nm).

## 3. Results and Discussion

**Figure 2:**
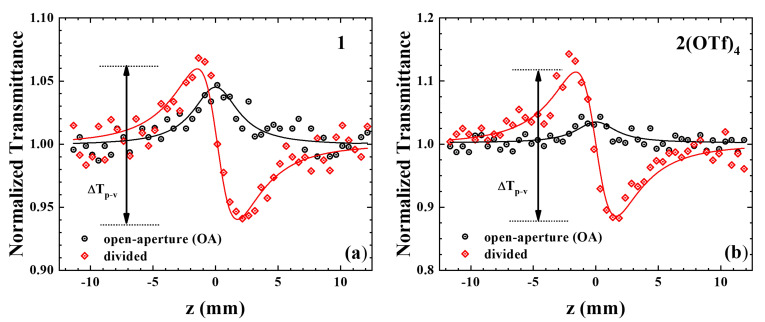
OA and “divided” Z-scans of BODIPYs (**a**) **1** (2.12 mM) and (**b**) **2(OTf)_4_** (0.423 mM) obtained under 12.5 and 31 MW/cm^2^, 4 ns, 532 nm laser excitation, respectively.

First, the experimental findings concerning BODIPYs **1** and **2(OTf)_4_** will be presented, as their characteristic absorption bands, located at 500 and 498 nm, respectively, are similarly distant from the laser excitation, i.e., at 532 nm. In Figure 2, some representative OA and “divided” Z-scans of **1** (2.12 mM) and **2(OTf)_4_** (0.845 mM) obtained under 532 nm laser excitation are presented. As shown, their OA Z-scans exhibit a transmittance maximum, indicative of saturable absorption (SA) behavior (corresponding to β < 0), while their corresponding “divided” Z-scans exhibit a pre-focal transmittance maximum followed by a post-focal transmittance minimum, suggesting self-defocusing behavior (corresponding to γ′ < 0). From the OA Z-scans performed under various incident laser intensities, for each different concentration solution of **1** and **2(OTf)_4_**, the corresponding NLO absorption coefficient *β* was determined.

**Figure 3:**
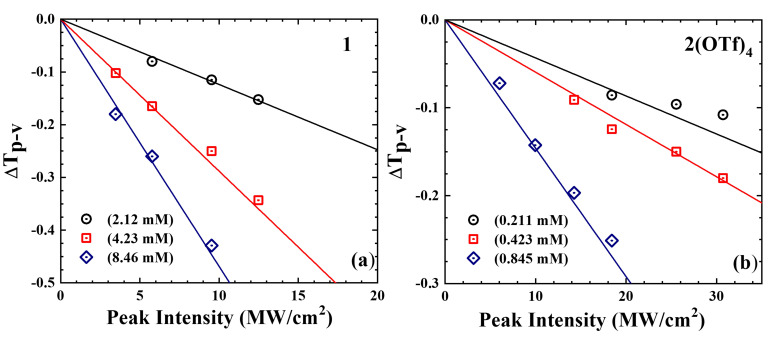
Variation in the ΔΤ_p-v_ values of different concentration solutions of BODIPYs (**a**) **1** and (**b**) **2(OTf)_4_** with the laser peak intensity, under 532 nm, 4 ns laser excitation.

In Figure 3, the variation in the values of the Δ*Τ_p-v_* parameter, obtained from the “divided” Z-scans of the different concentration solutions as a function of the incident laser intensity, is presented. From these plots, the values of the corresponding nonlinear refractive index parameter *γ*′ were determined, with its negative sign (self-defocusing behavior) being in agreement with the literature reports on relevant BODIPY compounds under similar exaltation conditions (see also Table 3). In all cases, the Δ*Τ_p-v_* values were found to vary linearly with the laser intensity, a behavior consistent with a third-order NLO response.

At this point, it is important to add that the solvent, DCM, measured under identical experimental conditions, exhibited a negligible NLO response. Therefore, the shown OA and “divided” Z-scans in Figure 2, as well as the slopes of the straight lines of Figure 3, reveal directly the NLO absorptive and refractive response of **1** and **2(OTf)_4_**.

In Table 2, the values of the NLO parameters *β* and *γ*′, determined from the Z-scan measurements, as well as the corresponding calculated values of *Imχ*^(3)^, *Reχ*^(3)^, and third-order nonlinear susceptibility *χ*^(3)^, and the second hyperpolarizability *γ*, of **1** and **2(OTf)_4_** under 4 ns, 532 nm laser excitation are presented. The determined values of all NLO parameters were found to scale with the concentration of the solutions. In addition, as can be seen, the χ^(3)^/c and second hyperpolarizability γ values of **2(OTf)_4_** were determined to be almost double that of **1**, revealing the larger NLO response of the former BODIPY.

Next, the results concerning BODIPYs **3**, **4**, and **BDPc** are presented. They were all found exhibiting very strong SA behavior (i.e., β < 0), and very weak self-defocusing (i.e., γ′ < 0) behaviour at the limits of the present experimental accuracy. The very strong SA response is attributed to the near-resonant excitation conditions being met, as their excitation at 532 nm is very close to their strong absorption bands located at 520, 516, and 517 nm, respectively. It should be remembered at this point that the “divided” Z-scan allows for the decoupling of the NLO refraction from the NLO absorption and the accurate determination of the nonlinear refractive index parameter γ′ when the sample exhibits non-negligible NLO absorption. Otherwise, the CA Z-scan is used to determine γ′. However, in the present case, the presence of such strong SA, being beyond the assumptions and limitations of the Z-scan technique, prohibits the decoupling of the two responses, preventing the determination of the nonlinear refractive response of the samples. To overcome this issue, several experiments were conducted, employing higher laser intensities (to enhance the signal of the “divided” Z-scans) and/or decreasing the concentration of the BODIPYs (to attenuate the SA response). Unfortunately, all efforts were unsuccessful, as in all cases, it was impossible to decouple the NLO refractive response from the corresponding NLO absorptive one. This situation prohibited the determination of the ΔΤ_p-v_ parameter and, therefore, the subsequent calculation of the nonlinear refractive index parameter γ′. As a result, only the nonlinear absorption coefficient β will be discussed next.

**Figure 4:**
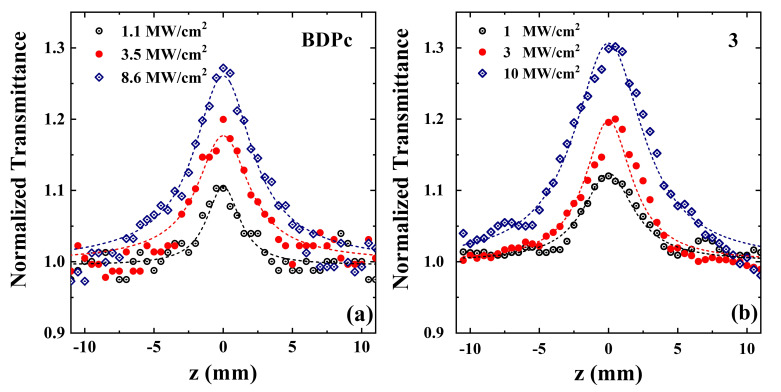
OA Z-scans of two DCM solutions of (**a**) **BDPc** (0.047 mM), and (**b**) **3** (0.082 mM), obtained under different laser intensities.

In Figure 4, some representative OA Z-scans of **BDPc** (0.047 mM) and **3** (0.082 mM), obtained under different incident laser intensities, are presented. The points represent the experimental data points, while the dashed lines correspond to the best fit of the OA Z-scans with Equation (1). As can be seen from these plots, the transmission maximum of the OA Z-scans (i.e., the SA response) was found to increase significantly with the laser intensity, and simultaneously exhibited significant broadening, evidencing the presence of important intensity saturation effects. This was also confirmed from the values of the *β,* which were determined using Equation (1). They were found to depend on the laser intensity used. In fact, all the solutions studied of **BDPc**, **3**, and **4** were found exhibiting similar intensity-dependent values of the NLO absorption coefficients *β*. It should be noted that the solutions of **BDPc**, **3**, and **4** were much more diluted (e.g., by an order of magnitude) compared to those employed for the study of BODIPYs **1** and **2(OTf)_4_**.

Under such absorption saturation conditions, the absorption coefficient α(I) is expressed as an intensity-dependent quantity as follows:

(6) $$\alpha \left(I\right)=\alpha_{0}+\beta I$$

where *α*_0_ (cm^−1^) is the linear absorption coefficient, *β* (cm/W) is the nonlinear absorption coefficient, and *I* (W/cm^2^) is the incident laser intensity.

In the case of SA, in particular, and assuming a three-level model for the description of the operation of a saturable absorber, and using the steady-state solution of the rate equations, the intensity-dependent absorption coefficient *a*(*I*) can be written as follows [33,34]:

(7) $$\alpha \left(I\right)=\frac{\alpha_{0}}{1+I/I_{s}}$$

where *I_s_* is the saturation intensity, defined as the intensity at which the intensity-dependent absorption coefficient *a*(*I*) drops to half of its value at the low-intensity regime.

Obviously, the lower the value of *I_s_*, the more easily the saturation of the absorption is attained.

Then, using Equations (6) and (7), the following expression of the intensity-dependent nonlinear absorption coefficient *β*(*I*) can be obtained:

(8) $$\beta \left(I\right)=-\frac{\alpha_{0}/I_{s}}{1+I/I_{s}}$$

**Figure 5:**
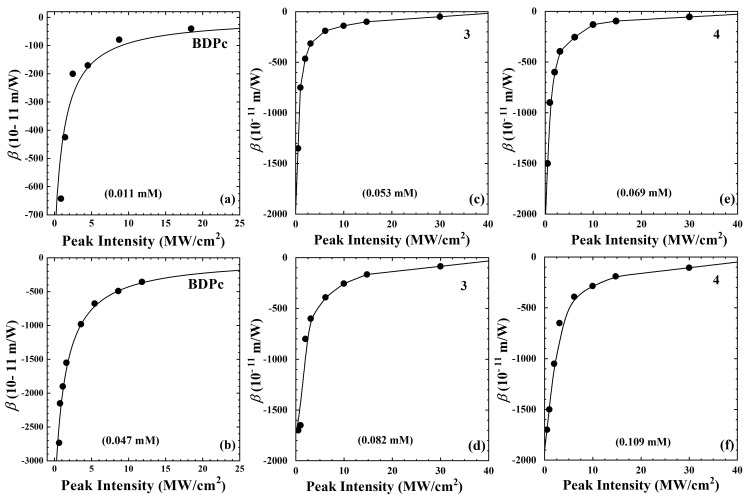
Variation in the NLO absorption coefficient *β* of different concentration solutions of (**a**,**b**) **BDPc**, (**c**,**d**) **3** and (**e**,**f**) **4**, with the laser peak intensity under 4 ns, 532 nm excitation.

From the Z-scan experiments, the determined NLO absorption coefficients *β* of all the different concentration solutions of **BDPc**, **3** and **4** were found to vary strongly with the incident laser intensity, all exhibiting similar intensity dependence, and confirming the presence of an intensity-saturated regime. In Figure 5, the variation in the β values of some different concentration solutions of **BDPc**, **3** and **4**, with the laser intensity under the 4 ns, 532 nm excitation, is shown as an example. It is interesting to note that the determined β values of all the solutions were found to vary importantly and very rapidly, within a very narrow window of laser intensities. So, in the case of **BDPc**, the β values were determined to vary by approximately an order of magnitude in the range of 1–3 MW/cm^2^, while in the case of **3** and **4**, the corresponding variation also had a similar magnitude and occurred in the range of 1–6 MW/cm^2^. Moreover, they were all observed reaching a plateau, as shown by the solid lines connecting the experimental data points (which are used as a guide for the eye).

Next, the saturation intensity, *I_s_*, values of **BDPc**, **3** and **4**, were determined by fitting the corresponding OA Z-scans of Figure 4, with the following relation:

(9) $$\frac{dI}{dz^{\prime }}=-\alpha \left(I\right)I$$

where *z*′ is the propagation distance of the laser beam in the sample.

They were determined to be 4.1, 6.3, and 5.1 MW/cm^2^ for the **BDPc**, **3** and **4**, respectively, with the **BDPc** having the lowest saturation intensity *I**_s_***.

Then, similar Z-scan measurements were performed for all the BODIPYs’ solutions, i.e., **1**, **2(OTf)_4_**, **3**, **4**, and **BDPc**, under infrared laser excitation conditions (i.e., 4 ns, 1064 nm) and for a wide range of laser intensities. However, in this case, all BODIPYs were found to exhibit negligible NLO absorption and refraction for all of the different concentration solutions and laser intensities used. These experimental findings suggest that the two-photon absorption (TPA) of the present BODIPYs is insignificant, although its presence could be excluded based on their absorption spectra (see, e.g., Figure 1). As a matter of fact, there are some studies of similar BODIPYs reporting the observation of TPA in this spectral region; however, in these studies, femtosecond (fs) and/or picosecond (ps) laser pulses were used, and laser intensities of the order of GW/cm^2^ [24,35,36,37,38,39,40,41]. In fact, BODIPYs **1** and **2(OTf)_4_**, having been investigated recently by Z-scan under 35 ps, 532/1064 nm and 50 fs, 800 nm laser excitation conditions, were found to exhibit negligible NLO absorption under ps excitation, and significant reverse saturable absorption (RSA) under fs excitation, with the latter having been attributed to two-photon absorption (TPA) [41]. Interestingly, both BODIPYs were reported to exhibit self-defocusing behavior under both ps and fs excitation conditions, while their NLO response was negligible under 35 ps and 1064 nm, similarly to what has been observed in the present study in the case of 5 ns, 1064 nm excitation conditions.

In Table 3, the NLO parameters of some BODIPYs, having similar structures to those studied in the present work, and measured under similar excitation conditions (i.e., under ns excitation), and reported in the literature, are presented. The wavelength of the peak of the characteristic absorption band, λ^abs^_max_ (nm) of these BODIPYs, is also included in this table, together with the excitation conditions used for the measurements of the NLO properties. As can be seen from these results, the BODIPYs can exhibit either saturable absorption (SA) or RSA behavior, depending on the detuning of the laser excitation wavelength from their characteristic absorption band. One should remember that the latter can be shifted considerably, depending on the nature and the number of the attached peripheral substituents to the BODIPY core, as has been reported in several other works, e.g., [42,43,44,45,46,47,48,49,50]. In fact, it is this situation that allows the large tunability of the BODIPYs’ photophysical properties, and therefore the tunability of their linear and nonlinear optical properties. So, as can be seen from Table 3, in all cases where excitation was taking place near the peak of the characteristic absorption band of the BODIPYs, SA behavior was observed, while when this band was shifted (e.g., because of functionalization of the BODIPY core), the NLO absorptive response was turned to RSA.

**Table 3:** NLO parameters of some other similar BODIPYs reported in the literature.

BODIPY	λ^abs^_max_ (nm)	Excitation	NLO Parameters	Reference
** 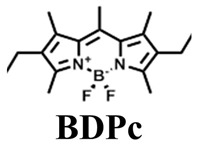 **	520	532 nm, 4 ns	*β* = −2.7 × 10^−8^ m/W *I_s_* = 4.1 MW/cm^2^	[this work]
** 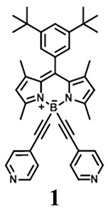 **	500	532 nm, 4 ns	*β* = −9.0 × 10^−9^ m/W *γ*′ = −5.1 × 10^−16^ m^2^/W *χ*^(3)^ = 8 × 10^−11^ esu *γ* = 4.9 × 10^−30^ esu	[this work]
** 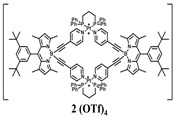 **	498	532 nm, 4 ns	*β* = −0.7 × 10^−9^ m/W *γ*′ = −1.2 × 10^−16^ m^2^/W *χ*^(3)^ = 1.6 × 10^−11^ esu *γ* = 8.9 × 10^−30^ esu	[this work]
** 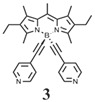 **	516	532 nm, 4 ns	*β* = −1.7 × 10^−8^ m/W *I_s_* = 6.3 MW/cm^2^	[this work]
** 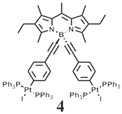 **	517	532 nm, 4 ns	*β* = −1.7 × 10^−8^ m/W *I_s_* = 5.1 MW/cm^2^	[this work]
** 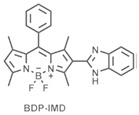 **	515	532 nm, 5 ns	*β* = −0.7 × 10^−11^ m/W *γ*′ = 9.5 × 10^−19^ m^2^/W *χ*^(3)^ = 3.8 × 10^−13^ esu	[42]
** 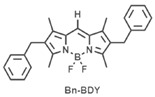 **	527	532 nm, 5 ns	*β* = −2.7 × 10^−11^ m/W *γ*′ = 4.9 × 10^−19^ m^2^/W *χ*^(3)^ = 12.2 × 10^−13^ esu	[43]
** 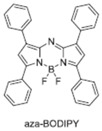 **	663	532 nm, 3 ns	*β* = 3.1 × 10^−10^ m/W	[44]
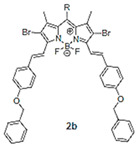	663	532 nm, 10 ns	*β* = 2.2 × 10^−9^ m/W *γ* = 9.8 × 10^−30^ esu	[45]
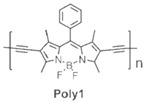	640	532 nm, 4 ns	*β* = 2.0 × 10^−10^ m/W *γ*′ = 1.7 × 10^−15^ m^2^/W	[46]
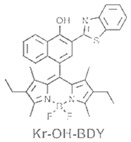	532	532 nm, 5 ns	*β* = −4.7 × 10^−12^ m/W *γ*′ = 2.3 × 10^−19^ m^2^/W *χ*^(3)^ = 1.1 × 10^−12^ esu	[47]

## 4. Conclusions

In summary, in the present work, the third-order NLO properties of four BODIPY dyes, namely **1**, **2(OTf)_4_**, **3**, and **4**, as well as the BODIPY core unit, **BDPc**, were investigated by Z-scan under 4 ns, both visible (532 nm) and infrared (1064 nm), laser excitation. All the BODIPY derivatives, under 532 nm excitation, were found to exhibit significant NLO response of comparable magnitude, if not larger, to that of other similar BODIPYs reported in the literature, while they presented negligible response under 1064 nm excitation. More specifically, BODIPYs **1** and **2(OTf)_4_** exhibited important SA (β < 0) and self-defocusing (γ′ < 0) responses, while **3**, **4**, and **BDPc** presented significantly stronger SA behavior and negligible self-defocusing, not being possible to be measured accurately due to the presence of the very strong saturable absorption. In this case, the saturation intensities *I_s_* were determined. They were determined to be of the order of 5 MW/cm^2^. The present experimental results demonstrate the significant role of the tuning of the photophysical characteristics of the BODIPYs and/or the excitation conditions (e.g., the excitation wavelength) on the tailoring of their nonlinear optical properties. The present observations highlight the important role of BODIPYs’ functionalization, underscoring their critical role for optimizing the NLO performance of the BODIPY derivatives for photonic and optoelectronic applications, making them unique candidates for delivering largely tunable linear and nonlinear optical properties.

## Figures and Tables

**Table 1:** Molecular weights and photophysical characteristics of the studied BODIPYs. For the assembly of **2(OTf)_4_**, the ε_max_ refers to the molar absorptivity/BODIPY subunit.

BODIPY	MW	α_0_/c (cm^−1^/mM)	λ^abs^_max_ (nm)	ε_max_ (mol^−1^ L cm^−1^)	Φ_f_	λ^fl^_max_ (nm)	k_f_ (×10^8^ s^−1^)	τ (ns)
**BDPc**	318.21	107	520	78,200	0.88	538	1.44	6.10
**1**	602.62	0.64	500	94,160	0.93	508	1.83	5.06
**2(OTf)_4_**	3016.55	0.17	498	94,160	0.95	508	1.59	5.94
**3**	484.44	60	516	78,400	0.83	534	1.38	5.98
**4**	2173.56	59	517	77,300	0.86	534	1.24	6.91

**Table 2:** Determined values of the  NLO parameters β, γ′, Imχ^(3)^, Reχ^(3)^, χ^(3)^, and second hyperpolarizability γ of **1** and **2(OTf)4**, under 4 ns, 532 nm laser excitation.

BODIPY	*c* (mM)	*β* (×10^−9^ m/W)	*γ*′ (×10^−16^ m^2^/W)	*Imχ*^(3)^ (×10^−11^ esu)	*Reχ*^(3)^ (×10^−11^ esu)	*χ*^(3)^ (×10^−11^ esu)	*χ*^(3)^/*c* (×10^−11^ esu/mM)	*γ* (×10^−30^ esu)
**1**	8.46	−9.0	−5.1	−5.0	−6.6	8.0	1.0 ± 0.1	4.9 ± 0.3
	4.23	−4.4	−2.9	−2.3	−3.7	4.4		
	2.12	−2.0	−1.6	−1.0	−2.1	2.0		
**2(OTf)_4_**	0.845	−0.7	−1.2	−0.4	−1.5	1.6	1.9 ± 0.3	8.9 ± 0.8
	0.423	−0.5	−0.4	−0.3	−0.5	0.6		
	0.211	−0.3	−0.3	−0.2	−0.4	0.5		

## Data Availability

The data presented in this study are available on request.

## References

[1] Ulrich G., Ziessel R., Harriman A. (2008). The chemistry of fluorescent bodipy dyes: Versatility unsurpassed. Angew. Chem. Int. Ed..

[2] Boens N., Leen V., Dehaen W. (2012). Fluorescent indicators based on BODIPY. Chem. Soc. Rev..

[3] Loudet A., Burgess K. (2007). BODIPY dyes and their derivatives: Syntheses and spectroscopic properties. Chem. Rev..

[4] Liu S., Shi Z., Xu W., Yang H., Xi N., Liu X., Zhao Q., Huang W. (2014). A class of wavelength-tunable near-infrared aza-BODIPY dyes and their application for sensing mercury ion. Dye. Pigment..

[5] Pereira N.A.M., Pinho e Melo T.M.V.D. (2014). Recent Developments in the Synthesis of Dipyrromethanes. A Review. Org. Prep. Proced. Int..

[6] Singh-Rachford T.N., Haefele A., Ziessel R., Castellano F.N. (2008). Boron Dipyrromethene Chromophores: Next Generation Triplet Acceptors/Annihilators for Low Power Upconversion Schemes. J. Am. Chem. Soc..

[7] Benstead M., Mehl G.H., Boyle R.W. (2011). 4,4-Difluoro-4-bora-3a,4a-diaza-s-indacenes (BODIPYs) as components of novel light active materials. Tetrahedron.

[8] Li F., Yang S.I., Ciringh Y., Seth J., Martin C.H., Singh D.L., Kim D., Birge R.R., Bocian D.F., Holten D. (1998). Design, synthesis, and photodynamics of light-harvesting arrays comprised of a porphyrin and one, two, or eight boron-dipyrrin accessory pigments. J. Am. Chem. Soc..

[9] Khan T.K., Broring M., Mathur S., Ravikanth M. (2013). Boron dipyrrin-porphyrin conjugates. Coord. Chem. Rev..

[10] Diring S., Puntoriero F., Nastasi F., Campagna S., Ziessel R. (2009). Star-Shaped Multichromophoric Arrays from Bodipy Dyes Grafted on Truxene Core. J. Am. Chem. Soc..

[11] Ojida A., Sakamoto T., Inoue M.-A., Fujishima S.-H., Lippens G., Hamachi I. (2009). Fluorescent BODIPY-based Zn(II) complex as a molecular probe for selective detection of neurofibrillary tangles in the brains of Alzheimer’s disease patients. J. Am. Chem. Soc..

[12] Kamkaew A., Lim S.H., Lee H.B., Kiew L.V., Chung L.Y., Burgess K. (2013). BODIPY dyes in photodynamic therapy. Chem. Soc. Rev..

[13] Gabe Y., Urano Y., Kikuchi K., Kojima H., Nagano T. (2004). Highly sensitive fluorescence probes for nitric oxide based on boron dipyrromethene chromophores rational design of potentially useful bioimaging fluorescence probe. J. Am. Chem. Soc..

[14] Bessette A., Hanan G.S. (2014). Design, synthesis and photophysical studies of dipyrromethene-based materials: Insights into their applications in organic photovoltaic devices. Chem. Soc. Rev..

[15] Khan T.K., Sheokand P., Agarwal N. (2013). Synthesis and studies of aza-BODIPY-based π-conjugates for organic electronic applications. Eur. J. Org. Chem..

[16] Duran-Sampedro G., Agarrabeitia A.R., Garcia-Moreno I., Costela A., Bañuelos J., Arbeloa T., López Arbeloa I., Chiara J.L., Ortiz M.J. (2012). Chlorinated BODIPYs: Surprisingly efficient and highly photostable laser dyes. Eur. J. Org. Chem..

[17] Torre G., Vázquez P., Agulló-López F., Torres T. (2004). Role of structural factors in the nonlinear optical properties of phthalocyanines and related compounds. Chem. Rev..

[18] Yesodha S.K., Pillai C.K.S., Tsutsumi N. (2004). Stable polymeric materials for nonlinear optics: A review based on azobenzene systems. Prog. Polym. Sci..

[19] Garreau-de Bonneval B., Ching K.I.M.-C., Alary F., Bui T.-T., Valade L. (2010). Neutral d^8^ metal bis-dithiolene complexes: Synthesis, electronic properties and applications. Coord. Chem. Rev..

[20] Liu X., Zhang J., Li K., Sun X., Wu Z., Ren A., Feng J. (2013). New insights into two-photon absorption properties of functionalized aza-BODIPY dyes at telecommunication wavelengths: A theoretical study. Phys. Chem. Chem. Phys..

[21] Tekin S., Küçüköz B., Yilmaz H., Savinç G., Hayvali M., Yaglioglu H.G., Elmali A. (2013). Enhancement of two photon absorption properties by charge transfer innewly synthesized aza-boron-dipyrromethene compounds containing triphenylamine, 4-ethynyl-N,N-dimethylaniline and methoxy moieties. J. Photochem. Photobiol. A.

[22] Shi W.-J., Lo P.-C., Singh A., Ledoux-Rak I., Ng D.K.P. (2012). Synthesis and second-order nonlinear optical properties of push-pull BODIPY derivatives. Tetrahedron.

[23] Zheng Q., He G.S., Prasad P.N. (2009). A novel near IR two-photon absorbing chromophore: Optical limiting and stabilization performances at an optical communication wavelength. Chem. Phys. Lett..

[24] Adarsh N., Avirah R.R., Ramaiah D. (2010). Tuning photosensitized singlet oxygen generation efficiency of novel aza-BODIPY dyes. Org. Lett..

[25] Küçüköz B., Hayvali M., Yilmaz H., Uğuz B., Kürüm U., Yaglioglu H.G., Elmali A. (2012). Synthesis, optical properties and ultrafast dynamics of aza-boron-dipyrromethene compounds containing methoxy and hydroxy groups and two-photon absorption cross-section. J. Photochem. Photobiol. A.

[26] Xenogiannopoulou E., Medved M., Iliopoulos K., Couris S., Papadopoulos M.G., Bonifazi D., Sooambar C., Mateo-Alonso A., Prato M. (2007). Nonlinear optical properties of ferrocene- and porphyrin-[60]fullerene dyads. Chem. Phys. Chem..

[27] Imahori H., Sekiguchi Y., Kashiwagi Y., Sato T., Araki Y., Ito O., Yamada H., Fukuzumi S. (2004). Long-lived charge-separated state generated in a ferrocene-meso,meso-linked porphyrin trimer-fullerene pentad with high quantum-yield. Chem. Eur. J..

[28] Schuster D.I., Li K., Guldi D.M., Palkar A., Echegoyen L., Stanisky C., Cross R.J., Niemi M., Tkachenko N.V., Lemmetyinen H. (2007). Azobenzene-linked porphyrin-fullerene dyads. J. Am. Chem. Soc..

[29] Kaloudi-Chantzea A., Karakostas N., Raptopoulou C.P., Psycharis V., Saridakis E., Griebel J., Hermann R., Pistolis G. (2010). Coordination-Driven Self Assembly of a Brilliantly Fluorescent Rhomboid Cavitand Composed of Bodipy-Dye Subunits. J. Am. Chem. Soc..

[30] Kaloudi-Chantzea A., Karakostas N., Pitterl F., Raptopoulou C.P., Glezos N., Pistolis G. (2012). Efficient Supramolecular Synthesis of a Robust Circular Light—Harvesting Bodipy-dye Based Array. Chem. Commun..

[31] Kaloudi-Chantzea A., Martinou E., Seintis K., Karakostas N., Giastas P., Pitterl F., Oberacher H., Fakisb M., Pistolis G. (2016). Formation of a highly—Ordered Rigid Multichromophoric 3D Supramolecular Network by Combining Ionic and Coordination—Driven Self—Assembly. Chem. Commun..

[32] Aloukos P., Chatzikyriakos G., Papagiannouli I., Liaros N., Couris S. (2010). Transient nonlinear optical response of some symmetrical nickel dithiolene complexes. Chem. Phys. Lett..

[33] Boyd R.W. (1992). Nonlinear Optics.

[34] Sharma K.K., Rao K.D., Kumar G.R. (1994). Nonlinear optical interactions in dye-doped solids. Opt. Quantum Electron..

[35] Brédas J.L., Adant C., Tackx P., Persoons A. (1994). Third-order nonlinear optical response in organic materials: Theoretical and experimental aspects. Chem. Rev..

[36] Porrès L., Mongin O., Blanchard-Desce M. (2006). Synthesis, fluorescence and two-photon absorption properties of multichromophoric boron-dipyrromethene fluorophores for two-photon-excited fluorescence applications. Tetrahedron Lett..

[37] Zhao Z., Chen B., Geng J., Chang Z., Aparicio-Ixta L., Nie H., Chin Goh C., Guan Ng L., Qin A., Ramos-Ortiz G. (2014). Red Emissive Biocompatible Nanoparticles from Tetraphenylethene-Decorated BODIPY Luminogens for Two-Photon Excited Fluorescence Cellular Imaging and Mouse Brain Blood Vascular Visualization. Part. Part. Syst. Charact..

[38] Li L.-L., Li K., Li M.-Y., Shi L., Liu Y.-H., Zhang H., Pan S.-L., Wang N., Zhou Q., Yu X.-Q. (2018). BODIPY-Based Two-Photon Fluorescent Probe for Real-Time Monitoring of Lysosomal Viscosity with Fluorescence Lifetime Imaging Microscopy. Anal. Chem..

[39] Ren C., Deng X., Hu W., Li J., Miao X., Xiao S., Liu H., Fan Q., Wang K., He T. (2019). A near-infrared I emissive dye: Toward the application of saturable absorber and multiphoton fluorescence microscopy in the deep-tissue imaging window. Chem. Commun..

[40] Sevinc G., Kucukoz B., Elmali A., Hayvali M. (2020). The synthesis of -1, -3, -5, -7, -8 aryl substituted borondipyrromethene chromophores:Nonlinear optical and photophysical characterization. J. Mol. Struct..

[41] Aloukos P., Orfanos I., Dalamaras I., Kaloudi-Chantzea A., Avramopoulos A., Pistolis G., Couris S. (2022). Nonlinear optical response of some Boron-dipyrromethene dyes: An experimental and theoretical investigation. Mater. Chem. Phys..

[42] Thakare S.S., Screenath M.C., Chitrambalam S., Joe I.S., Sekar N. (2017). Non-linear optical study of BODIPY-benzimidazole conjugate by solvatochromic, Z-scan and theoretical methods. Opt. Mater..

[43] Mallah R.R., Mohbiya D.R., Screenath M.C., Chitrambalam S., Joe I.S., Sekar N. (2019). NLOphoric benzyl substituted BODIPY and BOPHY: A comprehensive linear and nonlinear optical study by spectroscopic, DFT and Z-scan measurement. Spectrochim. Acta Part A Mol. Biomol. Spectrosc..

[44] Frenette M., Hatamimoslehabadi M., Bellinger-Buckley S., Laoui S., Bag S., Dantiste O., Rochford J., Yelleswarapu C. (2014). Nonlinear optical properties of multipyrolle dyes. Chem. Phys. Lett..

[45] Ngoy B.P., May A.K., Mack J., Nyokong T. (2019). Effect of bromination on the optical limiting properties at 532 nm of BODIPY dyes with p-benzyloxystyryl groups at the 3,5-positions. J. Mol. Struct..

[46] Zhu M., Jiang L., Yuan M., Liu X., Ouyang C., Zheng H., Yin X., Zuo Z., Liu H., Li Y. (2008). Efficient Tuning Nonlinear Optical Properties: Synthesis and Characterization of a Series of Novel Poly(aryleneethynylene)s Co-Containing BODIPY. J. Polym. Sci. Pol. Chem..

[47] Mallah R.R., Mohbiya D.R., Screenath M.C., Chitrambalam S., Joe I.S., Sekar N. (2019). Non-linear optical response of meso substituted dipyrromethene boron difluoride dyes: Synthesis, photophysical, DFT and Z scan study. Opt. Mater..

[48] Divyasree M.C., Shiju E., Dijo P., Chandrasekharan K. (2019). ZnSe-BODIPY hybrid system for nonlinear optical switching applications. Mater. Chem. Phys..

[49] Kulyk B., Taboukhat S., Akdas-Kilig H., Fillaut J.-L., Boughalebb Y., Sahraoui B. (2016). Nonlinear refraction and absorption activity of dimethylaminostyryl substituted BODIPY dyes. RSC Adv..

[50] Kulyk B., Taboukhat S., Akdas-Kilig H., Fillaut J.-L., Boughalebb Y., Sahraoui B. (2017). Tuning the nonlinear optical properties of BODIPYs by functionalization with dimethylaminostyryl substituents. Dye. Pigment..

